# Prevalence and associated factors of postpartum anxiety among Latvian women: a cross-sectional study

**DOI:** 10.1192/j.eurpsy.2026.10648

**Published:** 2026-07-31

**Authors:** S. Cīpare, M. Lazareva, L. Renemane, L. Rubene - Kesele, V. V. Vinogradova, L. Ķīse, K. Šulte, A. Bērziņa, E. Rancāns

**Affiliations:** 1 Psychiatry and Narcology; 2 Obstetrics and Gynaecology, Rīga Stradiņš University, Riga, Latvia

## Abstract

**Introduction:**

Postpartum anxiety is a mental health concern with adverse effects on maternal well-being, infant development, and family functioning (Ayers et al. *Br J Psychiatry* 2023; 1:1–8). Symptom overlap with mood disorders and limited screening contribute to underrecognition (Barry et al. *JAMA* 2023; 329:2163–70). About 20% of women experience clinically significant anxiety during pregnancy and postpartum (Fawcett et al. *J Clin Psychiatry* 2019; 80:19m12789). No studies have examined postpartum anxiety in Latvia.

**Objectives:**

To assess the prevalence, severity, and associated sociodemographic and clinical factors of postpartum anxiety among women at their first outpatient gynaecology visit, 4–6 weeks after childbirth.

**Methods:**

This cross-sectional study included all consecutive women aged ≥18 years attending Riga Maternity Hospital, Latvia’s largest outpatient clinic, for a routine gynaecologist visit 4–6 weeks postpartum. Anxiety was measured with the Generalized Anxiety Disorder-7 scale (GAD-7) with a cut-off score of 8 chosen, based on sensitivity/specificity analysis. Sociodemographic (age, BMI, ethnicity, education, residence, employment, family income) and clinical/reproductive factors (children <18 years, breastfeeding, abortion history, delivery mode, assisted reproduction, perinatal complications, intrapartum interventions, chronic and gynaecological illness, psychiatric history, abuse during pregnancy, sleep quality, infant temperament) were collected via medical records and questionnaire. Associations were tested with chi-square.

**Results:**

A total of 272 women (mean age 30.66 ± 5.59 years) were assessed. Of these, 82% (n=223) had GAD-7 <8, while 18% (n=49) scored ≥8. Higher anxiety severity (GAD-7 ≥8) was significantly associated with maternal complications during/after delivery (p=0.009), older maternal age (p=0.013), gynaecological illness (p=0.019), history of mood disorder (p=0.026), and emotional abuse (p=0.044). Borderline associations were observed for stressful life events before/during pregnancy (p=0.076) and breastfeeding (p=0.097). No significant associations were found with BMI, residence, ethnicity, smoking, alcohol use, physical abuse, reproductive history, or other clinical factors (all p>0.05).

**Conclusions:**

Postpartum anxiety was common, affecting nearly one in five women. Key risk factors included older maternal age, gynaecological illness, maternal complications, prior mood disorder, and emotional abuse, with possible contributions from stressful life events and breastfeeding. These findings highlight the need for routine postpartum anxiety screening in Latvia.

**Disclosure of Interest:**

None Declared

